# Investigating a novel multiplex proteomics technology for detection of changes in serum protein concentrations that may correlate to tumor burden

**DOI:** 10.12688/f1000research.24654.2

**Published:** 2020-11-13

**Authors:** Annie He Ren, Ioannis Prassas, Antoninus Soosaipillai, Stephanie Jarvi, Steven Gallinger, Vathany Kulasingam, Eleftherios P. Diamandis

**Affiliations:** 1 Department of Laboratory Medicine and Pathobiology, University of Toronto, Toronto, Ontario, Canada; 2 Lunenfeld-Tanenbaum Research Institute, Mount Sinai Hospital, Toronto, Ontario, Canada; 3 Department of Pathology and Laboratory Medicine, Mount Sinai Hospital, Toronto, Ontario, Canada; 4 Pancreatic Surgical Oncology Program, University Health Network, Canada, Toronto, Ontario, Canada; 5 Department of Clinical Biochemistry, University Health Network, Toronto, Ontario, Canada

**Keywords:** proteomics, ELISA, multiplex, immunoassay, pancreatic cancer, protein technologies

## Abstract

**Background:** To account for cancer heterogeneity, we previously introduced the concept of “personalized” tumor markers, which are biomarkers that are informative in subsets of patients or even a single patient. Recent developments in various multiplex protein technologies create excitement for the discovery of markers of tumor burden in individual patients, but the reliability of the technologies remains to be tested for this purpose. Here, we sought to explore the potential of a novel proteomics platform, which utilizes a multiplexed antibody microarray, to detect changes in serum protein concentration that may correlate to tumor burden in pancreatic cancer.

**Methods:** We applied the Quantibody® Human Kiloplex Array to simultaneously measure 1,000 proteins in sera obtained pre- and post-surgically from five pancreatic cancer patients. We expected that proteins which decreased post-surgery may correlate to tumor burden. Sera from two healthy individuals, split into two aliquots each, were used as controls. To validate the multiplexed results, we used single-target ELISA assays to measure the proteins with the largest serum concentration changes after surgery in sera collected pre- and post-surgically from the previous five patients and 10 additional patients.

**Results:** The multiplexed array revealed nine proteins with more than two-fold post-surgical decrease in at least two of five patients. However, validation using single ELISAs showed that only two proteins tested displayed more than two-fold post-surgical decrease in one of the five original patients. In the independent cohort, six of the proteins tested showed at least a two-fold decrease post-surgery in at least one patient.

**Conclusions:** Our study found that the Quantibody® Human Kiloplex Array results could not be reliably replicated with individual ELISA assays and most hits would likely represent false positives if applied to biomarker discovery. These findings suggest that data from novel, high-throughput proteomic platforms need stringent validation to avoid false discoveries.

## Introduction

Cancer manifests through alterations in the expression and interaction of proteins, which taken together gives rise to hallmarks such as unchecked cellular proliferation and metastasis ^1^. Many studies have thus tried to dissect the cancer proteome, with the long-standing goal of finding biomarkers that predict disease status. However, there has been limited success in bringing new cancer protein biomarkers to the clinics ^2, 3^, partly due to limitations of the popular and robust technique used for proteomics - mass spectrometry (MS). Despite vast progress in MS-based methods over the past decade, the dynamic range of assays, especially in complex mixtures such as serum, may still lack the sensitivity to detect rare tumor-related proteins in biological fluids ^4, 5^. When it comes to sensitivity, there is perhaps no better tool than the enzyme-linked immunosorbent assay (ELISA). The technique however is restricted in multiplexing power due to limited availability of antibodies and cross reactivity. Recently, innovative ELISA-based microarrays overcame this problem by using nanofluidics to bridge multiplexing with unforeseen sensitivity, and as a result can reasonably measure thousands of proteins simultaneously ^6– 9^. We can now leverage such high throughput and resolution to study the cancer proteome, even in single patients.

Protein biomarker research has long focused on a one-size-fits-all approach, where the utility of biomarkers is largely based on how well they perform in the majority of patients of a cancer type. However, the existing mindset surrounding biomarker discovery has recently been challenged by an increasing knowledge on a vastly heterogeneous cancer landscape. With more and more genomics studies showing that tumor heterogeneity exists between patients, within metastatic and primary sites and even within the primary tumor ^10– 13^, it has become evident that past research may have overlooked the array of distinct tumor markers that may exist in each patient. Searches for new cancer biomarkers that work for most patients may thus be in vain, and a shift to profiling tumor-related proteins in individuals is warranted. In the light of this, our study seeks to test a novel concept, which we had previously introduced and coined as “rare” or “personalized” tumor markers ^14^. These biomarkers may not be very informative for the majority of patients of a cancer type, but may nevertheless be highly precise and sensitive at predicting tumor response to therapy and relapse in a small subset (10–15%) of patients, or even in a single patient ^14^. In the future, a comprehensive panel of such personalized tumor markers would allow for rapid and high throughput screening of blood-based samples using clinically trusted immunoassays, in order to identify the most informative biomarkers tailored to monitor therapeutic response and relapse in each patient ^14, 15^. This notion is analogous to the routinely used molecular approach, for which whole genome sequencing is used to identify actionable mutations for individual patients for therapy selection. In an age of precision medicine, our vision holds remarkable potential for advancing individualized cancer treatment by personalizing tumor surveillance in each patient to determine the optimal trigger points for switching therapy or initiating second-line treatment – especially in the numerous cancer types where traditional biomarkers fall short.

Here, we aim to take the first step toward examining the promise of new and exciting multiplexed protein technologies for identifying changes in serum proteins that could correspond to tumor burden in individual patients, using pancreatic cancer as a model. Pancreatic cancer is the fourth-leading cause of cancer deaths, with pancreatic ductal adenocarcinoma (PDAC) being the most lethal and common form (making up 90% of all cases) ^16, 17^. CA 19-9 is currently used for monitoring treatment efficacy, but it cannot detect PDAC early and is not expressed in patients who lack the Lewis antigen (about 10% of the Caucasian population) ^18, 19^. CA 19-9 can also be elevated in many disorders such as other gastrointestinal cancers and pancreatitis ^18, 19^. With an improved selection of new chemotherapeutics and immunotherapies on the horizon ^17, 20^, it is more critical than ever to assess which patient populations would benefit from initiating a new therapy, and when personalized tumor markers which closely monitor therapeutic efficacy and relapse could aid in guiding future treatment strategies and improve survival outcome on a patient-to-patient basis.

In this study, we used the high-throughput, multiplexed immunoassay, Quantibody® Human Kiloplex Array, to concurrently quantify 1,000 inflammation-related proteins (including CA 19-9) in the serum of five PDAC patients obtained pre- and post-tumor resection (n=10). As crosstalk between inflammation-related molecules is a key component of tumorigenesis ^21– 23^, we theorized that proteins which drastically drop in level following optimal tumor debulking surgery may be correlated to the change in tumor burden in the patients. These proteins were then quantified in a larger cohort of serum collected from 15 PDAC patients pre- and post-surgery (n=30) using commercially available ELISAs, in order to validate the reliability, accuracy, and reproducibility of the multiplexed array results. Our findings provide preliminary insights into the utility and challenges of applying emergent multiplexed proteomics instruments in identifying changes in serum protein levels in cancer.

## Methods

### Sample collection

Serum samples from PDAC patients were retrospectively obtained from the University Health Network BioBank with approval by the Research Ethics Board of University Health Network, Toronto, Canada (Approval number 10-0591). Informed consent forms outlining the serum collection process and research study were signed by patients prior to sample collection. Samples were stored at -80°C prior to analysis. Sera from five PDAC patients collected within one week before surgery and at four weeks after surgery were analyzed (n=10). One serum sample from a normal male and one from a normal female, each split into two identical aliquots (n=4), were used as controls.

Serum samples from another 10 PDAC patients collected within one week pre-surgery and at four weeks post-surgery, along with the previous cohort, were tested for validation (n=30). Sera from a normal male and female (n=2) were used as controls.

### Quantibody® Human Kiloplex Array Technology

This multi-analyte immunoassay offered by RayBiotech uses nanotechnology to combine 25 nonoverlapping arrays, essentially performing 1,000 sandwich ELISAs simultaneously. The microfluidics system boasts absolute quantification with good precision (intra-assay CV = 7–10%; inter-assay CV = 10–15%) and sensitivity (pg/mL). The biological relevance of the 1,000 molecules tested (available online at https://www.raybiotech.com/human-kiloplex/) is mostly inflammation-related, including cytokines, tumor markers, and transcription factors among others.

In each array, standards with predetermined concentrations were assayed alongside the samples to provide a standard curve. The samples were evaluated in technical quadruplicates, and the disease and control status were undisclosed to the technical personnel. Each sample was diluted two-fold to a final volume of 3 mL for use in all 25 arrays. The workflow, described in the manufacturer’s manual, is akin to that of a sandwich ELISA. In brief, capture antibody was bound to the glass surface. After blocking and sample incubation, nonspecific binding was washed away, and a biotin-labeled detection antibody was then added. Next, streptavidin-conjugated Cy3 equivalent dye was added, and the resulting fluorescent signals were read via a microarray laser scanner (GenePix 4200A, Molecular Dynamics, Sunnyvale, CA, USA). Q-Analyzer software (Raybiotech) was used to compute an absolute quantification by calculating the average of the quadruplicate values, and accounting for intra- and inter-slide normalization.

### ELISA procedures

For all ELISAs, PDAC sera were tested in singleton due to limited sample volume, while standards were tested in duplicate. All the commercial, single-target ELISAs used in this study were validated with spike-and-recovery and linearity-of-dilution assessments using human serum as described in the manufacturers’ manuals. We also internally optimized the manufacturer’s protocol for each ELISA kit to ensure linearity-of-sample in serum samples obtained from female and male healthy controls. The following sections describe the optimized protocols that were used for measuring each protein. DuoSet IC ELISA kits (R & D Systems, Minneapolis, MN, USA) were used to measure serum levels of CEACAM-1 [Catalogue number (Cat #) DY2244] and IL-17RA (DY177), according to the manufacturer’s protocol. In brief, the assay was performed at room temperature (RT) with three washes between steps. First, 96-well microtiter plates were coated with capture antibody (4 µg/mL) and incubated overnight. Plates were blocked for 1 h before samples (1:6 dilution for CEACAM-1; 1:3 for IL-17RA) and standards were loaded and incubated for 2 h. Biotinylated detection antibody (100 ng/mL) was added and incubated for 2 h. Streptavidin-HRP was then loaded and incubated for 20 min in the dark. Enhanced K-Blue® TMB substrate (Neogen, Lexington, KY, USA) was next added, followed by 20 min incubation in the dark. Finally, the fluorescent signal was visualized by adding 2N H _2_SO _4_ and then measuring with the Wallac EnVision 2103 Multilabel Reader (Perkin Elmer, Waltham, MA, USA).

The DuoSet IC ELISA kit was also used to quantify PD-L2 (Cat # DY1224) serum levels according to manufacturer’s instructions with the following changes. Plates were not blocked. Samples (1:6 dilution) and standards were prepared in 6% BSA at 50 μl/well. Then, a 1:1 mixture of Assay Buffer A (60 g/L BSA, 37 g KCl, 25 ml/L normal mouse serum, 100 ml/L normal goat serum and 10 g/L bovine IgG in 50 mM Tris, 0.005% (v/v) Tween-20, pH 7.8) and 6% BSA was added at 50 μl/well before a 2 h incubation. Detection antibody was prepared in Assay Buffer B, where buffer content was the same as Assay Buffer A but minus KCl.

Serum levels of DSCAM (Cat # ELH-DSCAM-1), GATA-4 (ELH-GATA4-1), C1QTNF9 (ELH-C1QTNF9-1), CREG1 (ELH-CREG-1), and CRISP2 (ELH-CRISP2-1) were measured using RayBio® ELISA kits (RayBiotech) following the manufacturer’s protocol. In brief, the assay was performed at RT with four washes between the following steps. Pre-coated plates were blocked prior to loading samples (1:2 dilution) and standards, followed by a 2.5 h incubation. Biotinylated detection antibody was then loaded and incubated for 1 h. Next, streptavidin-HRP was added and incubated for 45 min. TMB substrate, followed by 30 min incubation in the dark, and 2N H _2_SO _4_ were added, and finally the fluorescence signal was read using the microplate reader.

### Data mining

We mined the Human Protein Atlas for the nine candidate proteins to study the mRNA and/or protein expression in pancreatic cancer. For each protein, we focused on examining the Cell Atlas for RNA expression in different cell types, as well as the Pathology Atlas which provides data on RNA and protein expression in different cancer types.

### Statistical analysis

Statistical analyses were performed using the GraphPad Prism software (version 4.02). Pearson correlation coefficients were computed to evaluate intra-assay and inter-individual variation. Shapiro-Wilks test was used to assess data normality. A two-tailed paired t-test was performed for proteins where data was normally distributed, while Wilcoxon test was used otherwise. P-values of less than 0.05 were considered to be statistically significant.

## Results

### Validation of Quantibody® Array Technology

**Figure 1.**
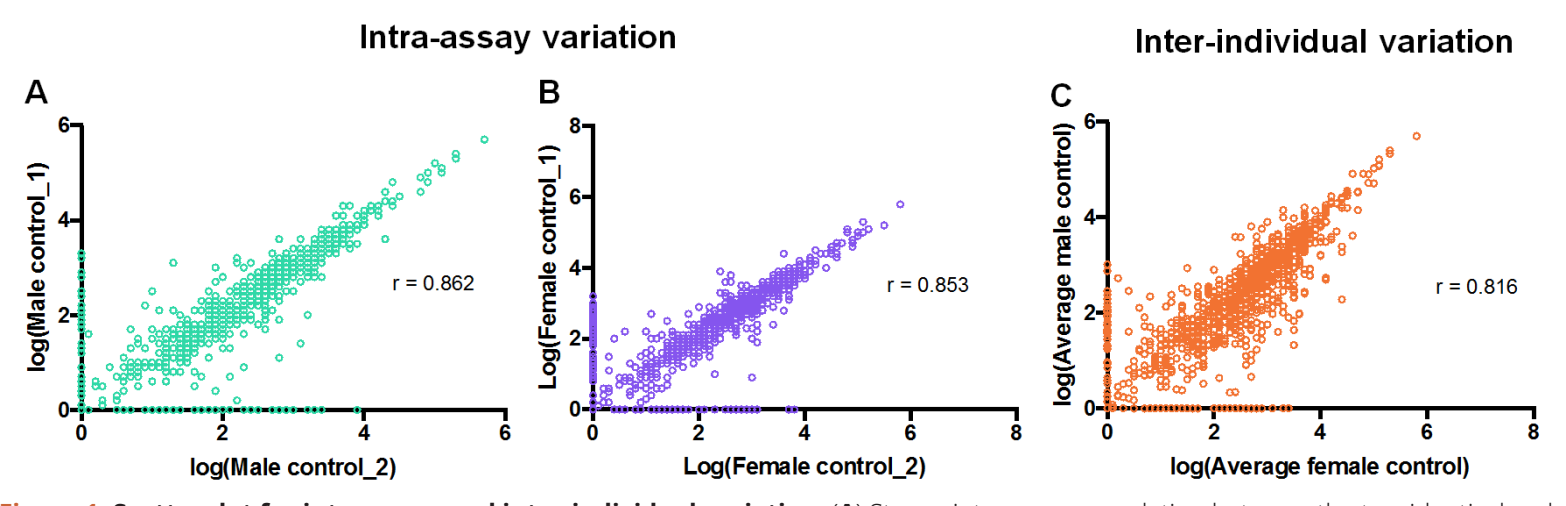
Scatterplot for intra-assay and inter-individual variation. ( **A**) Strong intra-assay correlation between the two identical male control samples (aliquot 1 vs. aliquot 2) that were assayed in a blinded fashion ( *P*<0.001). Similarly, ( **B**) shows strong correlation between the two identical female control samples ( *P*<0.001). ( **C**) Strong correlation between the average value of the two male control samples and that of the two female control samples ( *P*<0.001). Pearson correlation coefficient (r) was used for analysis.

We first sought to assess the reliability of the Quantibody® Human Kiloplex Array using scatterplot analysis ( Figure 1). Concentrations of the 1,000 proteins analyzed were log10 transformed to best visualize the large range of values. Intra-assay variability was assessed by testing two identical aliquots of one male and one female control sample ( Figure 1A, B). The Pearson correlation coefficients (r) for intra-assay variability between the two aliquots of the male ( Figure 1A) and female control sample ( Figure 1B) were 0.862 ( *P*<0.001) and 0.853 ( *P*<0.001) respectively, signifying overall good reproducibility of this assay. The inter-individual variability in the healthy controls was also assessed. For each male and female control, we computed an average value for the two aliquots, and visualized the correlation between the average values through a scatterplot ( Figure 1C). The Pearson correlation coefficient in this case was 0.821 ( *P*<0.001), denoting minimal inter-individual variability.

**Figure 2.**
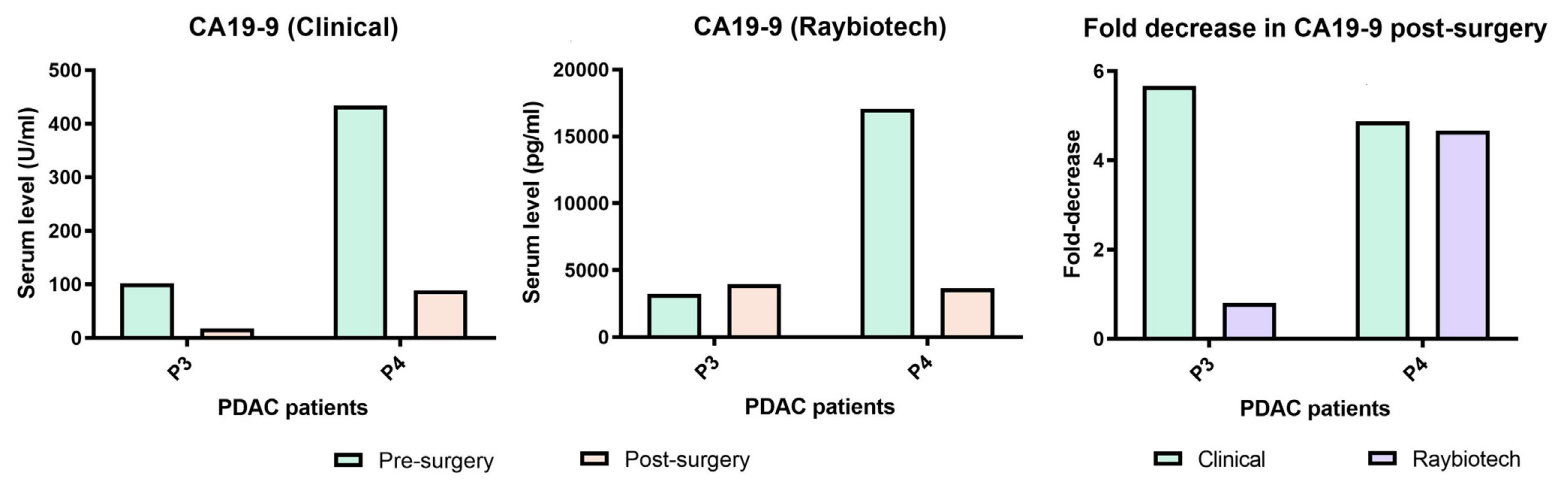
Comparison of CA 19-9 values obtained by clinical assessment versus Quantibody® array. ( **A**) Clinical value of CA 19-9 before and after surgery in two PDAC patients. ( **B**) CA 19-9 values obtained from the Quantibody® multiplexed array (Raybiotech) in the same patients. ( **C**) The fold decrease seen in the two PDAC patients according to clinical and multiplexed data.

Since the Quantibody® array includes the classical PDAC biomarker, CA 19-9, we also compared the post-surgical fold change in serum CA 19-9 level reported by the clinic to those obtained from the Quantibody® array in two of the five patients, where clinical data was available ( Figure 2A, B). Following tumor resection, patient 3 (P3) had a 5.7-fold decrease, while patient 4 (P4) saw a 4.7-fold decrease in CA 19-9 according to clinical assessment ( Figure 2C). On the other hand, the Quantibody® array data displayed a post-surgical 0.8- and 4.7-fold decrease in CA 19-9, in P3 and P4, respectively ( Figure 2C).

Raw data for each figure, in addition to Quantibody® output data, are available as *Underlying data* ^24^.

### Identifying proteins with largest changes in serum concentration after surgery based on multiplexed protein expression analysis

**Figure 3.**
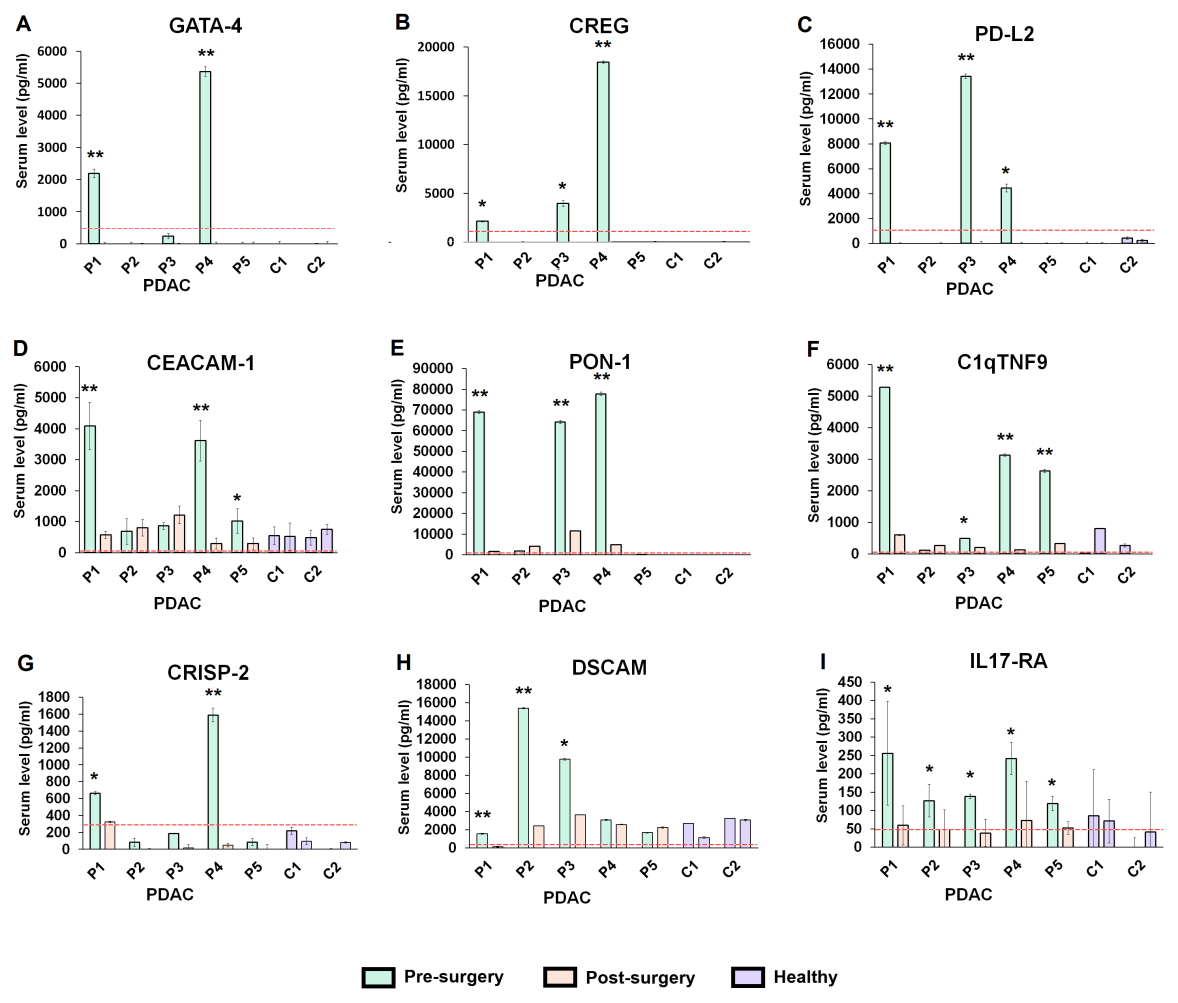
Multiplexed array results for the proteins that decreased in serum level following surgery in pancreatic cancer (PDAC) patients. Bar graphs depict normalized serum level of proteins in five PDAC patients (P1-P5), before and after surgery, and in two controls (C1 and C2). Red dashed line depicts limit of detection. Error bars represent 95% confidence intervals based on the technical quadruplicate values. * Post-surgical decrease of at least two-fold. ** Post-surgical decrease of at least five-fold. ( **A**– **C**) Levels of CREG, GATA-4 and PD-L2 dropped to undetectable levels in patients who expressed the protein prior to surgery. ( **D**– **F**) PON1, C1qTNF9 and CEACAM-1 levels decreased by at least five-fold in patients who highly expressed the protein pre-surgery. ( **G**– **I**) DSCAM, IL-17RA and CRISP-2 levels decreased by at least two-fold in at least two of five patients.

For the 1,000 proteins tested by the Quantibody® Kiloplex Array, we calculated the fold change in serum concentration from the pre-surgical to post-surgical value for each patient. Post-surgical values that were below the limit of detection were normalized to equal the value of limit of detection. We found nine proteins that were decreased by at least two-fold in at least two of the five patients ( Figure 3). Three proteins (GATA-4, CREG, and PD-L2) showed a drastic drop to undetectable levels after surgery in patients who initially expressed them before surgery ( Figure 3A–C). Serum levels of CEACAM-1, PON1, and C1qTNF9 decreased by at least five-fold in patients who highly expressed the protein before surgery ( Figure 3D–F). Finally, DSCAM, IL-17RA and CRISP-2 levels dropped by at least two-fold in patients who showed high levels before surgery ( Figure 3G–I).

**Figure 4.**
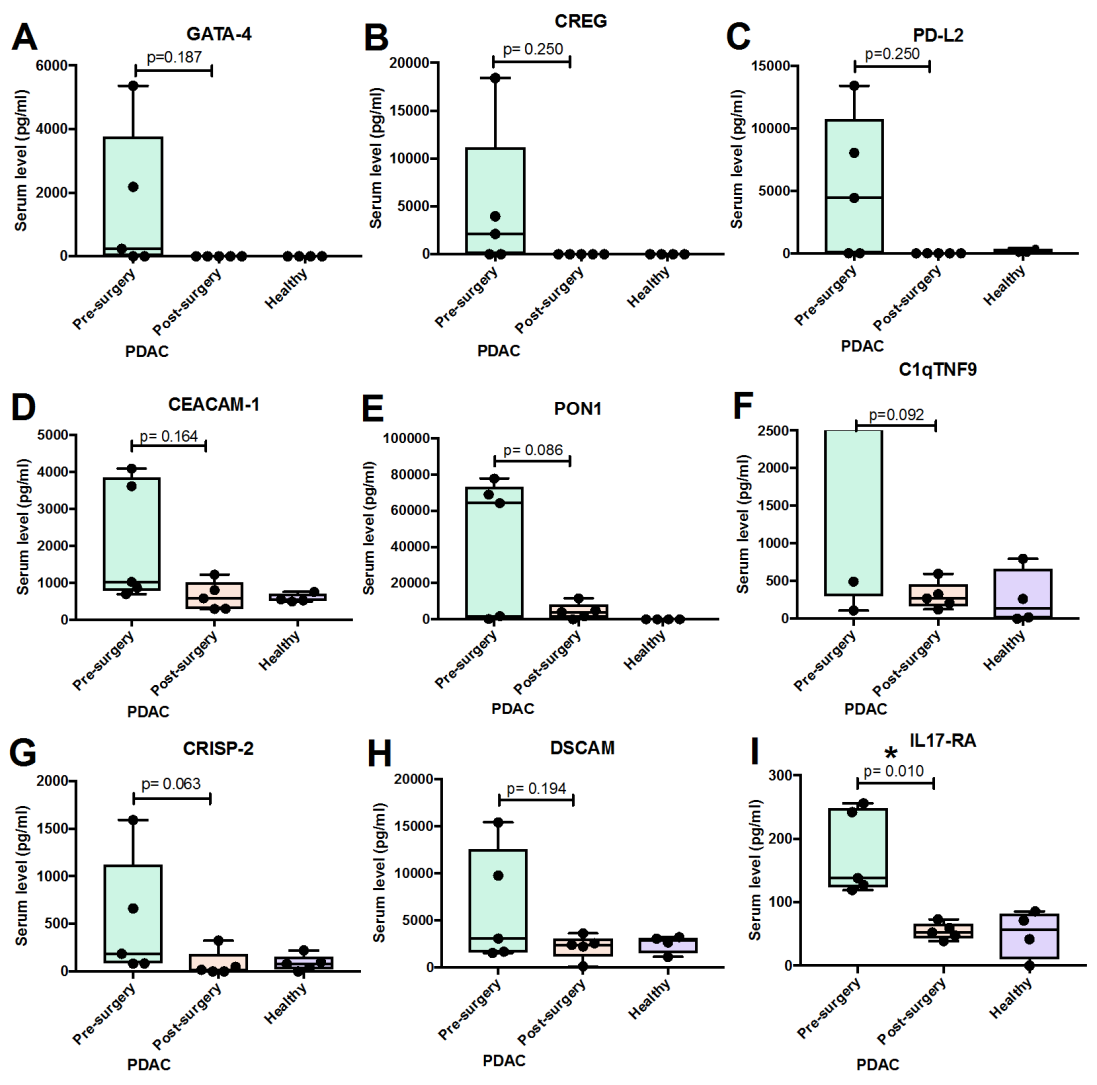
Group analysis of proteins that showed decrease in serum level in individual pancreatic cancer (PDAC) patients collected before and after surgery. Wilcoxon or paired T-test was used to collectively compare paired patient samples obtained pre-surgery (n=5) and post-surgery (n=5). The healthy group consists of all aliquots from the controls (n=4). Only IL-17RA levels were significantly reduced post-surgery (P<0.01, paired T-test).

Subsequently, we performed a group analysis by comparing the collective patient samples obtained pre-surgically (n=5) to samples obtained post-surgically (n=5) for the nine proteins ( Figure 4). Out of the nine proteins selected from the multiplex assay results, only IL-17RA levels dropped significantly post-surgery when comparing the patient cohort as a whole (P=0.01, paired t-test) ( Figure 4I).

### Validation of changes in protein serum levels for PDAC with ELISA

**Figure 5.**
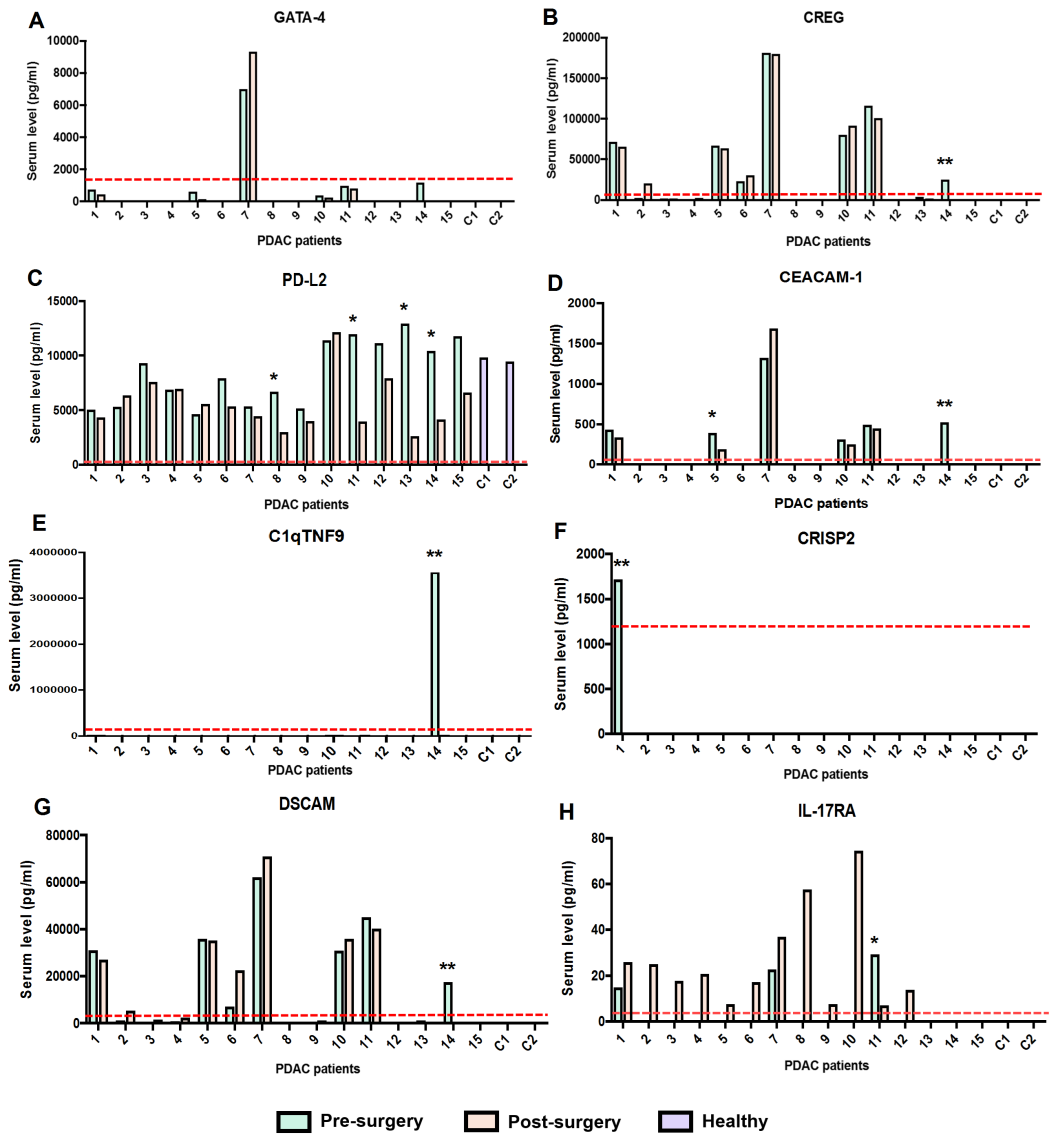
Validation of proteins that decreased in serum level in pancreatic cancer (PDAC) patients using single target ELISA assays. Bar graphs depict serum level of the proteins in fifteen PDAC patients (P1-P15), before and after surgery, and in two controls (C1 and C2). Red dashed line depicts limit of detection. * Post-surgical decrease of at least two-fold. ** Protein level dropped to undetectable level following surgery.

We aimed to perform single-target ELISA assays to quantitatively measure the expression levels of the nine proteins that was selected from the multiplex assay results in a larger cohort of PDAC patients. Sera collected pre- and post-surgically from an independent cohort of 10 PDAC patients were used (n=20). In order to compare data between the ELISA and Quantibody® array, we included sera from the original five PDAC patients (n=10, pre- and post-surgery). One of the proteins, PON1, was not tested as there was no reliable ELISA assay for quantifying in serum. Amongst the eight proteins assessed in the original patients (P1-P5), only CEACAM-1 and CRISP-2 showed a post-surgical fold decrease that aligned with the Quantibody® array data ( Figure 5). Specifically, P5 displayed a two-fold decrease in CEACAM-1, while P1 showed a drop in CRISP-2 to undetectable levels following tumor resection ( Figure 5D, F). In the independent cohort (P6-15), at least one patient showed a greater than two-fold decrease post-surgery in six proteins (CREG, PD-L2, CEACAM-1, C1qTNF9, DSCAM, and IL-17RA) ( Figure 5). However, this observed fold change requires further validation.

## Discussion

Cancer is not a homogeneous disease, but rather displays genetic and phenotypic variations between not only patients but even within a single tumor ^25, 26^. Considering the inter- and intra-heterogeneity of tumors, we previously introduced the concept of “rare” or “personalized” tumor markers, which are biomarkers that predict tumor load in small subsets of patients or even in a single patient ^14^. With the advent of multiplexed antibody-based microarrays, we can potentially search for such rare tumor markers in individual patients at never-before-seen depth, in a small volume (less than 1 mL) of blood-based sample. Here, we wanted to explore the feasibility of using a multiplexed proteomics platform to quantify serological proteins in pancreatic cancer that may be present at low concentrations (in the pg/mL range) and may change in serum level in correlation to changes in tumor burden after surgery. Pancreatic cancer, particularly the subtype PDAC, currently lacks appropriate biomarkers for detecting tumor presence early and sensitively ^16, 17, 19^. Patients may benefit from personalized biomarkers that sensitively monitor therapeutic response and recurrence in order to optimize treatment plans and improve patient outcome. With the emergence of various multiplex proteomics technologies, there is eagerness to identify personalized tumor markers in the serum of cancer patients. However, the first step to this long journey would be to test the feasibility and reliability of the new technology for accurately detecting changes in serum level of proteins in cancer patients in relation to a distinct change in tumor burden, such as the removal of the tumor through surgery. In this study, we applied the Quantibody® Human Kiloplex Array to determine the expression profiles of 1,000 proteins in serum of PDAC patients collected pre- and post-surgically. We postulate that proteins which decrease significantly in level after tumor resection may correspond to tumor burden in the patient, depending on the reliability and accuracy of the new multiplex technology at hand.

We first evaluated the reliability of the Quantibody® Kiloplex Array by examining the intra-assay variability. Samples from one healthy male and female, each split into two aliquots (n=4), were included as technical and biological controls. Although the overall correlation between the aliquots of male and female control samples was strong, many proteins showed zero value for one aliquot and high value for the other ( Figure 1A, B). This suggests that some proteins in this assay exhibited high technical variation even after normalization for intra- and inter-slide differences. In fact, the technical quadruplicate values obtained prior to normalization unveiled high inconsistency with apparent outliers for many proteins (data not shown, available as *Underlying data*) ^24^. A study that used a similar Quantibody® array to concurrently measure 174 cytokines in serum of ovarian cancer patients had seen very low variability, with the Pearson correlation coefficients for intra-slide and inter-slide reproducibility being 0.923 (P<0.001) and 0.899 (P<0.001) respectively ^27^. As the first to report the intra-assay variability of the Quantibody® Kiloplex Array, our findings suggest that this technology may lose reproducibility in some targets when scaled to 1,000 proteins. Additionally, we analyzed the inter-individual variability between the male and female control samples. Once again, the overall correlation was strong, but some proteins showed large variation in concentration between the two individuals ( Figure 1C). Since we are interested in finding tumor markers in single patients, any inter-individual variation is unlikely to affect the analysis. Finally, we compared the CA 19-9 values reported by the clinic with those obtained from the Quantibody® Kiloplex Array for two patients. Although both methods showed distinct post-surgical decreases in CA 19-9 in P4 ( Figure 2B, C), P3 did not show a decrease in CA 19-9 post-surgery compared to before surgery according to the Quantibody® data ( Figure 2A), which was not in concordance with the clinical data ( Figure 2C). This discrepancy may be due to either technical variation, day-to-day variation as the sera were taken at different time points, or a combination of both factors.

Delving into the selection of proteins with the largest change in serum level after surgery, we mainly took into account the fold change seen in protein level post-surgery compared to pre-surgery in each patient. This approach generated nine proteins whose serum concentration decreased by at least two-fold in at least two of the five patients ( Figure 3). Of the nine proteins that showed decline in serum level after surgery, GATA-4, PON1, CEACAM-1 and DSCAM have already been studied in pancreatic cancer and were found to be associated with immune suppression, aggressive phenotype, cancer progression and/or metastasis ^28– 31^. Remarkably, polymorphisms in the *PON1* gene have been associated with increased PON1 expression in a subset of pancreatic cancer patients ^30^. Our other protein that showed decrease in serum level after surgery, CREG (gastric cancer), IL-17RA (gastric/colorectal cancer), and PD-L2 (breast/liver cancer) have been associated with other cancers ^32– 35^. Furthermore, overexpression of C1qTNF9 has been proposed to activate aberrant AKT and MAPK signaling pathways in cancer cells ^33^. On the other hand, CRISP2 is mainly expressed in the testes and has not been studied in cancer ^36^. Additional mining of the Human Protein Atlas for the nine proteins further confirmed overexpression at the mRNA and/or protein level in a proportion of pancreatic cancer patients.

To validate the concentration changes we observed in the proteins using the multiplex assay, we used commercially available and verified single target ELISAs to accurately measure each protein in the sera of 15 PDAC patients taken before and after surgery. For comparison with the Quantibody® Kiloplex Array data, we included sera from the same five patients who were previously screened using the multiplexed assay. Of the eight proteins tested, a post-surgical fold decrease of at least two-fold, which would align with the multiplexed array results, was only observed in CEACAM-1 and CRISP2 in one patient each ( Figure 5D, F). As we used commercial, single-target ELISAs that were fully validated for reliable protein quantitation in human serum by the manufacturer and internally, it is unlikely that the results from the single-target ELISAs were erroneous. Furthermore, the antibodies used in the Raybio® ELISA kits for the proteins DSCAM, GATA-4, C1QTNF9, CREG1, and CRISP2 were obtained from RayBiotech and are the same antibodies used in the Quantibody® Kiloplex Array. However, as the antibodies used for CEACAM-1, IL-17RA, and PD-L2 in the Kiloplex Array are not reported by Raybiotech, we do not know whether the antibodies in the DuoSet IC ELISA kits from R & D Systems for these three proteins were the same. Nevertheless, our findings show that the protein quantitation data from the Kiloplex Array did not show concordance with those obtained from the verified, single-target ELISAs, even in the five Raybio® kits that used the same antibodies. This unfavorable outcome may suggest that highly multiplexed platforms may hinder the accuracy and reproducibility of measurements in some target proteins, and may suffer from high false discovery rate, where the observed fold change may reflect technical inconsistency as discussed in our evaluation of the intra-assay reproducibility. To date, seven studies have used similar Quantibody® arrays at smaller scales to measure a range of six to 320 proteins simultaneously in the serum of various diseases ^37– 40^. Notably, Green *et al.* used a Quantibody® array to concurrently examine the level of 10 cytokines in the serum of head and neck squamous cell carcinoma patients (n=101) taken before and after tumor treatment ^37^. The study found six cytokines that were significantly reduced post-treatment, however the results were neither validated via a different approach nor in an independent cohort ^37^. Only one other study published to date has used the Quantibody® Kiloplex Array, where Platonov *et al*. ** leveraged it to delineate the protein pathways resulting from *KISS1* gene expression in human breast cancer cell lines ^41^. Although they identified numerous secreted proteins that were disregulated in conditioned media upon *KISS1* activation, the results were not validated using a secondary analytical method ^41^. All in all, we are the first to employ the Quantibody® Kiloplex Array to simultaneously measure 1,000 proteins in sera of cancer patients. Moreover, we further attempted to validate the array results using a trusted independent approach. Our efforts suggest that the use of new multiplexed proteomics platforms, such as the Quantibody® Kiloplex Array, in biomarker discovery may still be nascent and pose significant questions in terms of precision and reproducibility. Nevertheless, a handful of revolutionary tools remains to be explored. Dionne *et al*. have aimed to compare the Quantibody® array with the magnetic bead-based MILLIPLEX® multiplex assay to simultaneously detect the levels of 40 cytokines in extracted tear samples, remarking that each method was superior at detecting a specific subset of cytokines ^42^. Furthermore, two studies have separately employed multi-analyte technologies (Proseek® proximity extension assay versus the ELISA-based Simple Plex™ assay) to assemble a panel of biomarkers for the early detection of ovarian cancer ^43, 44^. Data from the Proseek® assay for CA125 values (known biomarker for ovarian cancer) had displayed a strong correlation with those obtained from clinical assays ^43^. The study showed promise for multiplexed tools to replicate results obtained from established clinical assays, and to discover new candidate biomarkers in cancer ^43^.

## Conclusion

Limitations in MS-based techniques (which has been traditionally been used for proteomics) has instigated an explosion of novel, multiplexed proteomics technologies, each promising high precision and resolution while only using miniscule amounts of blood-based samples (<1 mL). However, the jury is still out for whether novel multiplexed technologies are accurate and sensitive enough for biomarker discovery in complex biological fluids. Although our study used a limited sample size and cannot conclusively determine the technical reliability of the Quantibody® Kiloplex Array, we observed a tentatively high rate of potential false positives using the Quantibody® Kiloplex Array for identifying changes in protein concentration in the serum of pancreatic cancer patients, where validation results using commercial, established single-target ELISAs did not show concordance with the Kiloplex Array data. Our findings from a small sample size suggest that while the influx of pioneering proteomics tools may bring excitement around a new era of personalized biomarker discovery, it may be best to corroborate results and findings through independent orthogonal techniques in order to minimize the risk of false discoveries. Studies that fail to validate the candidate biomarkers resulting from multiplex proteomics platforms using independent approaches may be unknowingly presenting a potentially high rate of false positives. Moving forward, our proposed emergent concept of developing a comprehensive panel of “rare” or “personalized tumor markers” seeks to challenge the existing mentality surrounding cancer biomarkers. Our study aimed to provide novel information for where proteomics and cancer biomarker research is going, and encourage future research on the feasibility of using pioneering proteomics platforms, outside of MS, for personalized cancer biomarker discovery. With further exploration of novel proteomics platforms and their promise in biomarker research, we envision that unveiling a truly precise and sensitive technology can make personalized proteomics a reality – where thousands of proteins can be precisely quantified using a drop of serum in order to identify the most informative personalized tumor marker for an individual patient.

## Data availability

### Underlying data

Harvard Dataverse: Investigating a novel multiplex proteomics technology for detection of changes in serum protein concentrations that may correlate to tumor burden. https://doi.org/10.7910/DVN/N9K3OM ^24^.

This project contains the following underlying data:

- F1000R_Raw data_5.19.20 (XLSX). (Raw data for the present study, arranged by Figure.)
- Mount Sanai Hospital Service Report (XLSX; 8 files). (Output files from Raybiotech ELISA assays; each file contains data for approximately 100 proteins.)

Data are available under the terms of the Creative Commons Zero "No rights reserved" data waiver (CC0 1.0 Public domain dedication).
