# Supplementary material for: Investigating a novel multiplex proteomics technology for detection of changes in serum protein concentrations that may correlate to tumor burden
Source: F1000Res. 2020 Nov 13;9:732. Originally published 2020 Jul 20. [Version 2] doi: 10.12688/f1000research.24654.2 (PMC7682495; doi:10.12688/f1000research.24654.2)
Supplement: Peer review history

# Peer review: Reviewer response for version 2

**DOI:** 10.5256/f1000research.30537.r74762

**Published:** 2020-11-20

**Authors:** Anna E Lokshin

**Affiliations:** 1 Department of Epidemiology, Pathology, Medicine, and Obstetrics,Gynecology and Reproductive Sciences, University of Pittsburgh, Pittsburgh, PA, USA

The authors had responded to the few critiques I had. The manuscript is now ready for publishing

Is the work clearly and accurately presented and does it cite the current literature?

Yes

If applicable, is the statistical analysis and its interpretation appropriate?

Partly

Are all the source data underlying the results available to ensure full reproducibility?

Yes

Is the study design appropriate and is the work technically sound?

Partly

Are the conclusions drawn adequately supported by the results?

Partly

Are sufficient details of methods and analysis provided to allow replication by others?

Yes

Reviewer Expertise:

Cancer biomarkers, multiplexed immunoassay, pancreatic cancer

I confirm that I have read this submission and believe that I have an appropriate level of expertise to confirm that it is of an acceptable scientific standard.

# Peer review: Reviewer response for version 1

**DOI:** 10.5256/f1000research.27195.r70938

**Published:** 2020-10-22

**Authors:** Anna E Lokshin

**Affiliations:** 1 Department of Epidemiology, Pathology, Medicine, and Obstetrics,Gynecology and Reproductive Sciences, University of Pittsburgh, Pittsburgh, PA, USA

In this study, the authors explored a highly multiplexed antibody microarray platform, Quantibody Human Kiloplex array to detect changes in serum protein concentrations in pancreatic cancer patients before and after surgery to identify biomarkers that correlate that may correlate with tumor burden. The study is important in that it found that the Quantibody® Human Kiloplex Array results could not be reliably replicated with individual ELISA assays and most hits would likely represent false positives if applied to biomarker discovery. However, to directly compare two immunoassays, the same antibodies should be used. Additionally, the conclusions are based on too few cases.

Is the work clearly and accurately presented and does it cite the current literature?

Yes

If applicable, is the statistical analysis and its interpretation appropriate?

Partly

Are all the source data underlying the results available to ensure full reproducibility?

Yes

Is the study design appropriate and is the work technically sound?

Partly

Are the conclusions drawn adequately supported by the results?

Partly

Are sufficient details of methods and analysis provided to allow replication by others?

Yes

Reviewer Expertise:

Cancer biomarkers, multiplexed immunoassay, pancreatic cancer

I confirm that I have read this submission and believe that I have an appropriate level of expertise to confirm that it is of an acceptable scientific standard, however I have significant reservations, as outlined above.

# Peer review: Reviewer response for version 1

**DOI:** 10.5256/f1000research.27195.r70939

**Published:** 2020-09-10

**Authors:** Georgios Pampalakis

**Affiliations:** 1 Department of Pharmacy, School of Health Sciences, University of Patras, Patras, Greece

It is well-established that cancer exhibits a high degree of heterogeneity. The authors recently introduced the concept of personalized tumor markers to study tumor burden in individual patients. Here they attempted to explore this idea using a new commercially available antibody microarray. This microarray is based on a nanotechnology-based microfluidic multiplex sandwich ELISA for 1000 protein analytes. This system was developed to overcome certain limitations of mass spectrometry applied in clinical diagnosis. The specific aim is to identify changes of proteins in the serum of individuals before and after surgery that can be used to monitor therapeutic response or relapse. These proteins may differ between different patients and could be specific for even a single patient.

 The microarray used was a commercially available platform and importantly the authors validated the findings with classical ELISA assays. The authors analyzed serum samples obtained pre- and post-surgery in 5 patients. They found that the commercially available assay exhibits good reproducibility but the results could not be fully replicated with classical ELISA indicating that future improvements in the microarray platform are necessary in order to use it in clinical diagnosis.

 The work is clearly presented, and the methods are sufficiently detailed. All conclusions are fully supported by the data.

 Some drawbacks of the study are presented by the authors.

 Overall the study is clear and important since it compares a commercial high-throughput assay for reproducibility and accuracy.

Is the work clearly and accurately presented and does it cite the current literature?

Yes

If applicable, is the statistical analysis and its interpretation appropriate?

Yes

Are all the source data underlying the results available to ensure full reproducibility?

Yes

Is the study design appropriate and is the work technically sound?

Yes

Are the conclusions drawn adequately supported by the results?

Yes

Are sufficient details of methods and analysis provided to allow replication by others?

Yes

Reviewer Expertise:

Clinical diagnosis, biochemistry, activity-based probes, organophosphates, proteases

I confirm that I have read this submission and believe that I have an appropriate level of expertise to confirm that it is of an acceptable scientific standard.

# Response

**Published:** 2020-09-23

**Authors:** Eleftherios P Diamandis

**Affiliations:** Mount Sinai Hospital, Toronto, Canada

We would like to thank Dr Pampalakis for the constructive feedback.
